# Carbon Material Hybrid Construction on an Aptasensor for Monitoring Surgical Tumors

**DOI:** 10.1155/2022/9740784

**Published:** 2022-05-10

**Authors:** Renyuan Ma, Subash C. B. Gopinath, Thangavel Lakshmipriya, Yeng Chen

**Affiliations:** ^1^Department of General Surgery, Yulin No. 2 Hospital, Yulin 719000, China; ^2^Faculty of Chemical Engineering Technology, Universiti Malaysia Perlis (UniMAP), Arau 02600, Perlis, Malaysia; ^3^Institute of Nano Electronic Engineering, Universiti Malaysia Perlis (UniMAP), Kangar 01000, Perlis, Malaysia; ^4^Centre of Excellence for Nanobiotechnology and Nanomedicine (CoExNano), Faculty of Applied Sciences, AIMST University, Semeling, Bedong, 08100 Kedah, Malaysia; ^5^Department of Oral & Craniofacial Sciences, Faculty of Dentistry, University of Malaya, 50603 Kuala Lumpur, Malaysia

## Abstract

Carcinoembryonic antigen (CEA) is a glycoprotein, one of the common tumor biomarkers, found at low levels in body fluids. Generally, overexpression of CEA is found in various cancers, including ovarian, breast, lung, colorectal, gastric, and pancreatic cancers. Since CEA is an important tumor biomarker, the quantification of CEA is helpful for diagnosing cancer, monitoring tumor progression, and the follow-up treatment. This research develops a highly sensitive sandwich aptasensor for CEA identification on an interdigitated electrode sensor. Carbon-based material was used to attach a higher anti-CEA capture aptamer onto the sensor surface through a chemical linker, and then, CEA was quantified by the aptamer. Furthermore, CEA-spiked serum was tested by using the immobilized aptamer, which was found to not affect the target validation. The limit of detection for CEA in PBS and serum is calculated from a linear regression graph to be 0.5 ng/mL with *R*^2^ values of 0.9593 and 0.9657, respectively, over a linear range from 0.5 to 500 ng/mL. This CEA quantification by the aptasensor can help diagnose various surgical tumors and monitor their progression.

## 1. Introduction

Tumor biomarkers have a great value for diagnosing and screening tumors and help enhance the efficacy of evaluation. Tumor biomarkers are substances that can be attributed to the development of carcinogenesis at different stages of cell progression [1, 2]. Common tumor biomarkers are sugar lipids, protein, microRNA, RNA, and DNA. Carcinoembryonic antigen (CEA) is a glycoprotein expressed during the development of human fetuses. After birth, the expression of CEA is inhibited and found to be at a much lower level in healthy adults [3]. At the same time, the overexpression of CEA has been found in various human surgical and nonsurgical cancers, including gastric, lung, ovarian, breast, pancreatic, and colorectal cancers [4–7]. Therefore, quantifying CEA levels helps significantly in diagnosing cancer and monitoring the therapeutic condition. Current CEA identification in immunoassays requires expensive instruments and complex procedures. Therefore, a highly sensitive, selective, cost-effective, and rapid CEA biosensor is mandatory to detect and quantify the level of CEA.

Various biosensing techniques have been developed for the identification of CEA in human serum, such as radioimmunoassay, mass spectrometry, and enzyme-linked immunosorbent assay [8, 9]. Most biosensing methods are time consuming, expensive, and require complex procedures. Thus, developing a method with simple, low sensitivity, sensitivity, and speed is needed for CEA quantification. We used an electrochemical method with a carbon nanomaterial-modified electrode to detect CEA levels. Nanomaterial-applied biosensors have attracted increasing attention in the fields of medicine and diagnosis. The use of nanosized materials has led to enhanced detection of target molecules at a lower detection limit with increased sensitivity, faster detection, and lower cost [10–13]. Furthermore, the larger surface area of the nanomaterials enables the bioreceptor immobilization of an enhanced amount. Common nanomaterials, such as gold, graphene-derived materials, silver, aluminum, and silica, are more popular for various biomedical applications [14–16]. A biosensor basically consists of four major paths: a bioreceptor, transducer, signal processer (convert electrical to desired signal), and a display interface. In biosensor applications, nanomaterials are primarily used as transducer materials, which help to enhance the electrical signal upon the binding of biomolecules on the sensor surface. Apart from that, nanomaterial-conjugated biomolecules improve the stability of the molecules with a higher number of immobilizations on the sensing surface and enhance the biomolecular interaction. In particular, one-, two-, and three-dimensional carbon-based micro/nanomaterials, such as carbon nanotubes, carbon quantum dots, graphene, nanodiamonds, nanofibers, and carbon nanohorns, have been effectively utilized in various biomedical fields due to their significant mechanical, electronic, thermal, optical, and physical properties [17–19]. In addition, carbon materials in electrochemical sensors have been successfully proven to show better sensitivity, higher selectivity, and biocompatibility to identify a wide range of biological and chemical biomolecules [20]. This research work used carbon material for surface functionalization of an anti-CEA aptamer attachment on a sensor surface and enhanced the electric signal upon binding of CEA with its aptamer.

Similar to antibodies, aptamers have been found to act as efficient detection probes for various biomolecules [21–23]. The aptamer is a recently emerged molecule generated from the randomized library of molecules by a SELEX method. Aptamers are single-stranded RNA or DNA molecules that can specifically bind to targets, including peptides, proteins, small molecules, carbohydrates, toxins, and whole cells [24, 25]. Aptamers form various structures due to their tendency to form single-stranded loops and helices. Aptamers bind with the target via their secondary/tertiary structures rather than the primary sequence, which helps to bind with high sensitivity and selectivity. Due to their high binding affinity, aptamers are a focus in the field of biosensors for early diagnosis [26–29]. Aptamers have been selected against a wide range of targets, including tumor biomarkers. Circulating oligonucleotides, such as RNA and DNA, are commonly used as probes to identify various diseases, including tumors. In this research, an aptamer-based sandwich assay was conducted with specific quantitative analysis with CEA.

## 2. Materials and Methods

### 2.1. Reagents and Biomolecules

CEA (human) and bovine serum albumin (BSA) were ordered from Sigma Aldrich (USA). Graphene, 1-ethyl-3-[3-dimethylaminopropyl] carbodiimide hydrochloride (EDC), and N-hydroxysuccinimide (NHS) were purchased from Sigma–Aldrich (USA). The following aptamer sequences were used as capture and detection molecules [30]. Capture aptamer: 3′-NH_2_-TTAACTTATTCGACCTAT-5′; detection probe: 3′-CCCATAGGGAAG TGGGGGA-5′. The fabricated interdigitated electrode (IDE) surface was obtained from Metrohm Company (Switzerland).

### 2.2. Activation of Carbon Material

The carbon material (graphene) (5 mg) was mixed with 50 mL of HCl and heated under reflux with stirring for 10 h. Then, the purified graphene was mixed with acids (1 : 3 v/v ratio of HNO_3_ : H_2_SO_4_) and sonicated for 10 h at room temperature. The product was further washed with distilled water until reaching neutral pH to obtain carboxylated graphene (COOH-graphene).

### 2.3. Fabrication of the COOH-Graphene-Aptamer Electrode Surface

The working IDE was first washed with deionized water and dried under nitrogen flow. Then, the surface was treated with KOH for 10 min and thoroughly washed again using distilled water. Furthermore, COOH-graphene was carefully drop cast onto the KOH-treated electrode. Then, the activation of the COOH group in graphene was conducted by treating the electrode with EDC-NHS for 10 min. After the activation process, 1 *μ*M anti-CEA aptamer (amine-terminated aptamer) was introduced onto IDE and incubated for 2 h. Subsequently, 1% BSA was drop cast onto the aptamer-modified electrode and kept for 1 h to cover the unbound sites. Finally, IDE was washed with PBS buffer to remove the excess reagents and biomolecules. The electrochemical response was recorded for each immobilization process.

### 2.4. Quantification of CEA by Aptamer-CEA-Aptamer Sandwich Assay

An aptamer-based sandwich assay was conducted to quantify the level of CEA. CEA concentrations ranging from 0.5 ng/mL to 1000 ng/mL were titrated and drop cast onto the anti-CEA aptamer electrode surface. Then, 1 *μ*M of anti-CEA aptamer (detection aptamer sequence) was drop cast onto all the CEA-immobilized surfaces individually. The electrochemical response was recorded for each CEA concentration, and then, the difference in the current value was plotted in an Excel worksheet to calculate the linear regression coefficient. From this graph, the detection limit for CEA was calculated.

### 2.5. Quantification of CEA in Human Serum

To identify the clinical feasibility of the developed aptasensor, experiments were conducted with CEA-spiked serum. CEA at concentrations ranging from 0.5 ng/mL to 1000 ng/mL spiked independently in diluted human serum, and the same experiments were conducted as described previously. The electrochemical responses were recorded for each concentration, and the current value was compared with CEA-spiked PBS (without serum).

The limit of detection was determined as the lowest concentration of an analyte (on the calibration line with low concentrations) and compared to the background signal (*S*/*N* = 3 : 1) and limit of detection = standard deviation of the baseline + 3*σ*. All measurements were performed by using a picoammeter (Keithley) over a voltage range of 0–2 V with sweep intervals of 0.1 V. The whole setup was kept at room temperature, and all surface modifications and interactions were performed under wet conditions, either using double distilled water (for chemical modification) or 10 mM phosphate buffered saline at pH 7.4 (for biological interaction). Washings with ten reaction volumes of the respective solutions were carried out before the measurements were taken.

## 3. Results and Discussion

**Figure 1:**
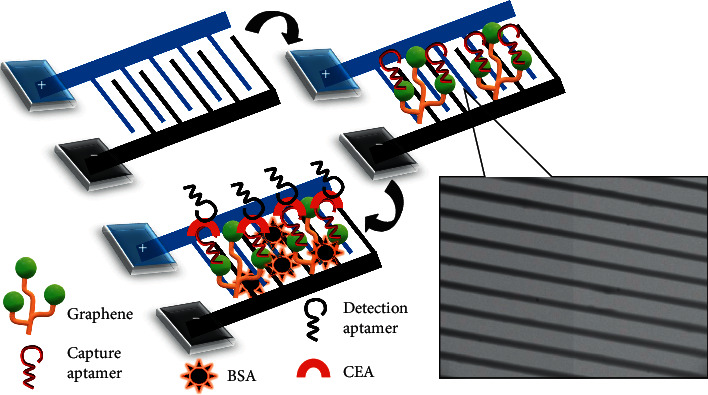
Graphical demonstration of the CEA aptasensor. A potassium hydroxide-treated electrode was constructed using COOH-activated graphene, and then, the anti-CEA aptamer was covalently immobilized onto the electrode surface through COOH with NH2. BSA was added to cover the excess activated surface, and then, the CEA was detected by the immobilized aptamer.

An aptasensor for the tumor biomarker CEA was generated on a graphene-modified interdigitated electrode (IDE) surface.  Figure 1 shows a graphical demonstration of the CEA aptamer-based assay. A potassium hydroxide-treated electrode surface was constructed with COOH-activated graphene and then activated with EDC-NHS to activate COOH groups on graphene to be converted into NH_2_. On the activated surface, the anti-CEA aptamer was covalently immobilized onto the electrode surface by COOH with NH_2_. Furthermore, BSA was added as a blocking agent to cover the excess activated surface. Finally, CEA was detected by a sandwich assay with the detection aptamer on the capture aptamer-constructed electrode surface. A two-dimensional graphene sheet was used for the surface modification on IDE. IDE was prepared on silica materials, and the dielectrode fingers were fabricated using aluminum. The fabricated dielectrode structure had a length of 8000 *μ*m and a width of 5000 *μ*m. The gap width among the 18 pairs of fingers created by the dielectrode was 100 *μ*m. Furthermore, the finger length was measured to be 4000 *μ*m, and the electrode thickness was 55 nm. The intactness of the received electrode surface was validated under a high-power microscope (Figure 1).

### 3.1. Imaging and EDX Analysis of Graphene

**Figure 2:**
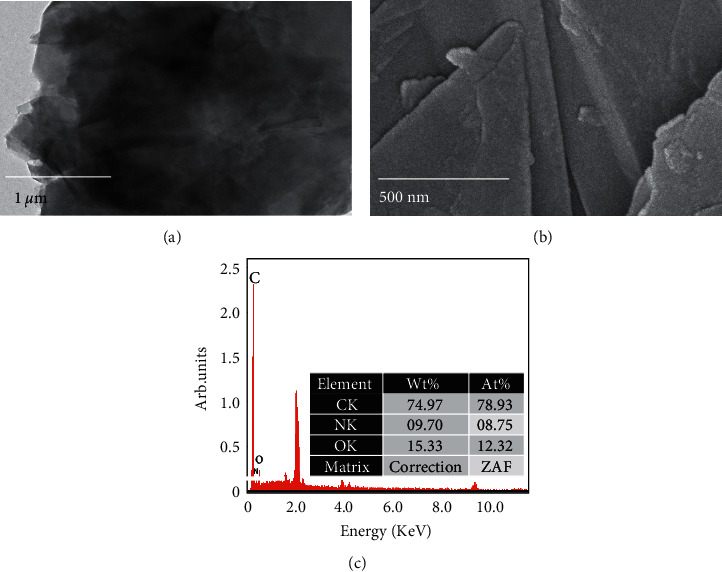
(a) FETEM image of graphene. (b) FESEM image of graphene. Images show a clear shaped flake with a uniform distribution. (c) EDX for graphene. The EDX result confirms that the major elements in graphene are carbon (74.97%) and O (15.33%).

The shape and size of the graphene image were analyzed by FESEM and FETEM observations (Figures 2(a) and 2(b)). As shown in figure, the graphene is observed to be a flake with a uniform distribution. The EDX result confirms the presence of the major elements of carbon (74.97%) and O (15.33%) in the graphene (Figure 2(c)).

### 3.2. Surface Functionalization with Anti-CEA Aptamer

**Figure 3:**
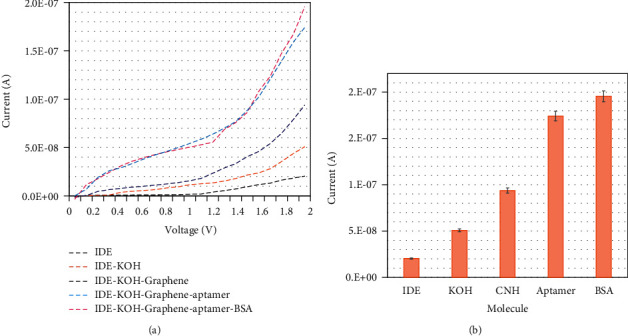
(a) Surface functionalization for anti-CEA aptamer immobilization. The electrochemical response for each process was recorded, and the immobilization was confirmed. This increment in response clearly indicates the attachment of the anti-CEA aptamer on the electrode surface. (b) Electrochemical response for anti-CEA aptamer attachment. The difference in response is observed to be much higher after the introduction of the aptamer on the sensing electrode surface, which confirms that the surface is modified with the anti-CEA aptamer process. The averaged values obtained from the replicates are displayed by the error bars.

Surface functionalization of the anti-CEA aptamer process was confirmed by an IDE sensor. The electrochemical response for each process was recorded and the immobilization process was confirmed (Figure 3(a)). The bare electrode shows a response of 2.04*E*−08 A, which is changed to 5.08*E*−08 A after treatment with KOH. The activated graphene further increases the response to 9.38*E*−08 A. This increased response clearly indicates the attachment of graphene on the electrode surface. Furthermore, the anti-CEA aptamer shows the highest electrochemical response of 1.74*E*−07 A. The difference in the response is observed to be much higher after the introduction of the aptamer on the sensing electrode, which confirms that the surface is modified with the anti-CEA aptamer (Figure 3(b)). Finally, BSA was added for blocking, and no significant response was noted because the activated sites on graphene are fully covered with anti-CEA aptamer. This result confirms that a higher number of anti-CEA aptamers is attached onto the electrode surface through graphene, which helps to lower the detection limit for CEA. Various studies have proven that a higher number of captured biomolecules immobilized with the right arrangement leads to a higher number of target molecules. In addition, graphene material-conjugated biomolecules are stable and help to increase the binding of biomolecules on the sensing surfaces. In this research, graphene is found to help to increase the anti-CEA aptamer immobilization on IDE and increase the electrochemical response upon the interaction of CEA with its aptamer.

### 3.3. Sandwich Assay of Aptamer-CEA-Aptamer on Graphene-Aptamer Immobilized Electrode Surface

**Figure 4:**
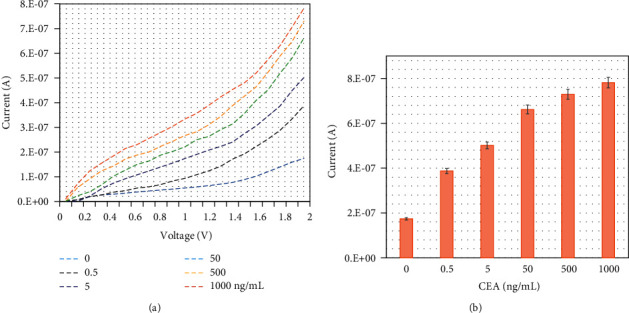
(a) CEA identification by sandwich assay with aptamer. Various concentrations of CEA were diluted in PBS and drop cast onto the anti-CEA aptamer-constructed electrode surface, and then, a fixed concentration of detection aptamer was added. A clear increase in the response was recorded for each CEA concentration. (b) Electrochemical response for CEA identification. The electrochemical response is increased by increasing the CEA concentration. The averaged values obtained from the replicates are displayed by the error bars.

Different concentrations of CEA were diluted in PBS and drop cast onto an anti-CEA aptamer-constructed electrode surface independently for CEA identification, and then, the fixed capture aptamer was added onto the surface. The lowest CEA concentration of 0.5 ng/mL was added to the electrode surface, and the current response was measured to be 3.87 *E*−07 A. The difference in current was determined to be 2.13 *E*−07 A, which confirms the binding of 0.5 ng/mL CEA with the immobilized aptamer (Figure 4(a)). By further increasing the CEA concentration to 5, 50, 500, and 1000 ng/mL, the responses were increased to 5.01 *E*−07, 6.62 *E*−07, 7.29 *E*−07, and 7.82 *E*−07 A, respectively. The electrochemical response was increased by increasing the CEA concentration (Figure 4(b)).

### 3.4. CEA Identification from Spiked Serum Samples

**Figure 5:**
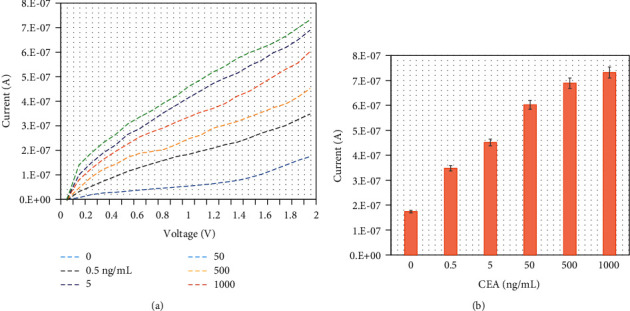
(a) CEA-spiked serum sample identification on the CNH-aptamer immobilized electrode surface. Various concentrations of CEA were diluted in serum and drop cast onto anti-CEA aptamer-constructed electrode surfaces for CEA identification. A clear increase in the response was recorded for each CEA concentration. (b) Electrochemical response for CEA-spiked serum identification. The electrochemical response was increased by increasing the CEA-spiked serum concentrations. The averaged values obtained from the replicates are displayed by the error bars.

Various concentrations of CEA were dissolved in 1 : 100 diluted human serum and drop cast onto an anti-CEA aptamer-constructed electrode surface for CEA identification. Initially, the lowest CEA concentration at 0.5 ng/mL was added to the electrode surface, and the current response was measured to be 3.48 *E*−07 A. The difference in current was determined to be *E*−07 A, which confirms the detection of 0.5 ng/mL CEA in the spiked serum sample assisted by the immobilized aptamer (Figure 5(a)). Furthermore, by increasing CEA concentrations in human serum to 5, 50, 500, and 1000 ng/mL, the responses were found to be increased to 4.51 *E*−07, 6.03 *E*−07, 6.89 *E*−07, and 7.3 *E*−07 A, respectively. Electrochemical responses were increased by increasing the CEA concentrations in the diluted serum (Figure 5(b)). This result shows the clear detection of CEA in human serum without any interferences.

### 3.5. Comparison of CEA Detection in Spiked PBS and Human Serum

**Figure 6:**
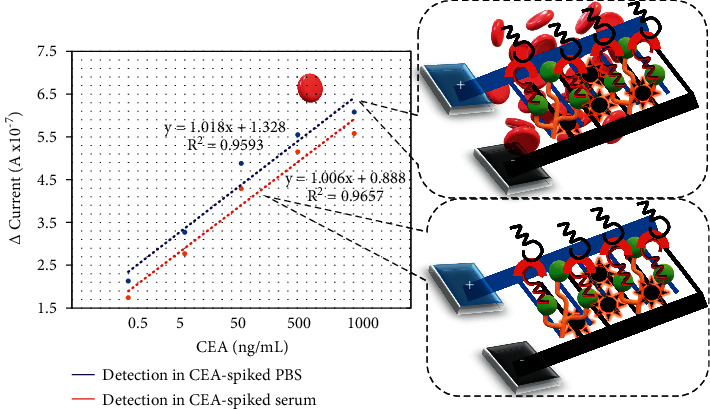
Limit of detection of CEA in PBS and human serum. Similar concentrations of CEA were diluted in PBS and human serum and detected on an anti-CEA aptamer-constructed IDE sensor. The difference in the response (from blank) was plotted by a linear regression graph. The limit of detection for CEA is found to be 0.5 ng/mL in both PBS and serum-spiked samples. Figure insets show the diagrammatical representations.

Similar concentrations of CEA (0.5–1000 ng/mL) were diluted in PBS or human serum and detected on the anti-CEA aptamer-constructed IDE sensor surface. The difference in response (from blank) was plotted on a linear regression graph and compared. As shown in Figure 6, CEA-spiked serum shows a slightly lower response than CEA-spiked serum in PBS, and the response of CEA at all concentrations increased with increasing CEA concentration. In addition, the limit of detection for CEA is found to be 0.5 ng/mL in both PBS and serum spiked samples. This result confirms the selective and effective identification of CEA in the biological sample.

## 4. Conclusion

Carcinoembryonic antigen (CEA) is a glycoprotein found to be a common biomarker for various tumors. Quantifying the level of CEA helps to diagnose the tumor and its condition at earlier stages. This research developed an aptamer-based sandwich assay for CEA identification on graphene-modified interdigitated electrodes. Higher anti-CEA capture-aptamer immobilization was achieved through graphene, and CEA was quantified by aptamer detection. Furthermore, CEA-spiked serum was found to show an increased electrochemical signal when CEA concentration was increased, which confirmed the detection of CEA in the biological fluid. The limit of detection for CEA in PBS and serum is calculated to be 0.5 ng/mL, with *R*^2^ values of 0.9593 and 0.9657, respectively. This CEA biosensor can help to quantify CEA at its lower level and diagnose various tumors.

## Data Availability

The data used to support the findings of this study are available from the corresponding author upon request.
